# Metagenomics approach to predict antibiotic resistance genes in sputum samples of adult people with cystic fibrosis: a pilot study

**DOI:** 10.1128/spectrum.02299-24

**Published:** 2025-11-05

**Authors:** Sonja van Scheijen, Anne H. Neerincx, Els J. M. Weersink, Josje Altenburg, Christof Majoor, Jacqueline E. van Muijlwijk-Koezen, Anke H. Maitland-van der Zee, Mahmoud I. Abdel-Aziz, On behalf of the Amsterdam Mucociliary Clearance Disease Research Group

**Affiliations:** 1 Department of Pulmonary Medicine, Amsterdam UMC, University of Amsterdam 26066 , Amsterdam, the Netherlands; 2 Innovations in Human Health and Life Sciences, Department of Chemistry and Pharmaceutical Sciences, Vrije Universiteit Amsterdam 1190, Amsterdam, the Netherlands; 3 Amsterdam Institute for Immunology and Infectious diseases, Infectious diseases 614286 , Amsterdam, the Netherlands; 4 Amsterdam Public Health, Personalized Medicine 1221, Amsterdam, the Netherlands; 5 AIMMS Molecular Research Accelerator, Faculty of Science, Vrije Universiteit Amsterdam 1190, Amsterdam, the Netherlands; 6 Department of Pediatric Pulmonology and Allergy, Emma Children's Hospital, Amsterdam UMC 26066 , Amsterdam, the Netherlands; The George Washington University School of Medicine and Health Sciences, Washington DC, USA

**Keywords:** antibiotic resistance, cystic fibrosis, antibiotic susceptibility testing, next-generation sequencing, metagenomics, artificial intelligence

## Abstract

Lung infections in people with cystic fibrosis (CF) cause lung damage, which is the leading factor in the morbidity and mortality of CF. Prescription of antibiotics to treat these infections is essential to maintain a higher quality of life and increase life expectancy. Determination of antibiotic susceptibility (ABS) is done by culture-dependent, phenotypic methods. These procedures take several days, while timely intervention is key. The analysis of antibiotic resistance genes by use of shotgun metagenomics might offer a time-sensitive alternative. Twenty people with CF with a homozygous Phe508del mutation provided 68 sputum samples during different visits over a period of roughly a year. After shotgun sequencing, the samples were analyzed using the deep learning tool deepARG. These results were compared with the results from routine ABS testing. The performance was determined by area under the curve-receiver operating characteristics (AUCROC) and sensitivity. Significant results were obtained for the following antibiotic classes: aminoglycoside (AUCROC = 0.81 [95% CI: 0.67–0.95, sensitivity = 73%]), cephalosporin (AUCROC = 0.70 [95% CI: 0.54–0.86, sensitivity = 95%]), and fluoroquinolone (AUCROC = 0.73 [95% CI: 0.56–0.89, sensitivity = 88%]). For other antibiotic classes, results were not significant. Using antibiotic class-specific cut-offs for positive reads of ARGs, a metagenomics approach potentially offers a culture-independent and more time-efficient manner to predict ABS for commonly prescribed antibiotic classes for sputum samples of adult people with CF. The use of metagenomics and artificial intelligence in clinical care will add to more personalized care for people with CF as well as better antibiotic stewardship.

**IMPORTANCE:**

Damage induced by lung infections in people with cystic fibrosis (CF) is the leading factor to the mortality and morbidity of CF. To treat bacterial infections, people with CF are prescribed antibiotics. Routine antibiotic susceptibility (ABS) testing relies on culture-dependent, phenotypic techniques. These take several days up to more than a week, while timely intervention is key. To bridge this time gap, physicians in CF care use patient history of ABS data to start antibiotics, with risk of resistance to it. This pilot study explores a time saving alternative: the possibility to predict antibiotic resistance genes using shotgun metagenomics and artificial intelligence. By quicker prediction of ABS, people with CF can receive more adequate care, which results in the possible prevention of chronic infections and contributes to antibiotic stewardship.

## INTRODUCTION

Lung infections in people with cystic fibrosis (CF) cause lung damage, which is the leading factor to the morbidity and mortality of CF (1–3). The most common causative agents of infection are *Pseudomonas aeruginosa* and *Staphylococcus aureus* (4). Prescription of antibiotics to treat these infections is essential to maintain a higher quality of life and increase life expectancy (5). Additionally, people with CF can be prescribed prophylactic antibiotics to attempt to prevent infection and are often on long-term antibiotics for chronic lung infections (6). The continued use of antibiotics increases the chance for developing antibiotic resistance, resulting in an increase of difficult-to-treat multidrug-resistant pathogenic strains in people with CF (7). Lastly, incorrect usage of antibiotics can lead to serious complications, such as toxicities affecting different organs, intolerances (8), antibiotic allergies (9), and drug-drug interactions (e.g., between cystic fibrosis transmembrane conductance regulator [CFTR] modulators and antibiotics [10]).

Routine clinical care for people with CF involves testing a sputum sample or cough swab for detection of pathogenic microbes roughly every 3 months (6, 11). Additionally, in case of a pulmonary exacerbation, additional detection of microbes is performed (12). The gold standard to detect these microbes is *in vitro* tests in which bacteria are cultivated on selected media (culture-dependent testing) (13). Detected microbes are subsequently tested for antibiotic susceptibility (ABS). Usually, this is done by *in vitro* so-called “phenotypic methods.” These methods depend on a visible inhibition of bacterial growth by the tested antibiotics (14).

This approach has its limitations. The most important limitation is time, as detecting pathogens and subsequent antibiotic susceptibility testing can span several days. These days can be critical in the treatment of pulmonary exacerbations (15). To timely treat infection, patients are often prescribed broad-spectrum antibiotics or antibiotics based on medical history while awaiting their results (11). This can add to the already introduced problem of antibiotic resistance in people with CF (7). The broad-spectrum antibiotic can lead to adverse reactions and have a negative effect on the general lung microbiome and the lung function (16).

Another limitation is that these methods depend on selected growth conditions and antibiotics. The CF lung microbiome is complex and dynamic, and also less well-known pathogens are present (13, 17), which are not always identified by culture-dependent testing (13), such as *Streptococcus milleri* and *Rothia* spp., which have been associated with pulmonary exacerbations (18, 19). This issue is further exemplified by the occurrence of culture-negative pulmonary exacerbations, where people with CF have a pulmonary exacerbation while cultures for CF-typical pathogens are negative (20). Lastly, the *in vitro* testing does not necessarily resemble the *in vivo* reaction of the patient (21, 22).

An alternative approach to culture-dependent testing could be a metagenomics approach. This approach entails sequencing the microbial genetic material present in a sample, thereby enabling broad characterization of the lung microbiome. Although it cannot detect all genetic materials due to technical and biological limitations (23, 24), this approach offers the possibility to develop relatively fast protocols (25) and is also culture independent. Antibiotic resistance in bacteria is mostly genetically encoded by so-called antibiotic-resistant genes (ARGs). Several bioinformatics tools to detect ARGs in metagenomics samples have been developed (26). Detecting ARGs from metagenomics data is increasingly used for scientific research; however, its usefulness in clinical settings has not been adequately explored to date (27). Although ARGs can be detected in patients’ samples, doubts exist whether these could reflect on the true antibiotic susceptibility phenotype (28, 29).

By improving antibiotic susceptibility testing, people with CF can have better treatment strategies by personalizing the selection of antibiotics in a timely manner. We hypothesize that culture-independent metagenomics allows timely prediction of ARGs that may be concordant with the antibiotic susceptibility phenotype as performed in the microbiology lab. We aim to demonstrate the feasibility of the use of metagenomics to predict the presence of ARGs in sputum samples of people with CF, contributing to future advancements in predicting antibiotic susceptibility phenotypes.

## MATERIALS AND METHODS

### Subjects and study design

This study was a longitudinal observational cohort study at the Amsterdam University Medical Center, location AMC. The study included 20 adult people with CF with a homozygous Phe508del mutation. Inclusion and exclusion criteria and study design are described previously in detail (30). The study received ethical approval by the ethical board of the Amsterdam UMC, and written consent was obtained from all patients.

### Sputum samples

Sputum samples were collected from patients at five different visits over the course of a year (roughly every 3 months), during the routine standard care as described previously in detail (30). Briefly, the first study visit’s samples were collected before the start of lumacaftor/ivacaftor, while the remaining four visits’ samples were collected after initiating the treatment. Sputum was obtained by asking the patient to cough deeply to collect sputum from the lower airways. If a patient was not able to produce sputum, a cough swab was taken following routine clinical care standards. Cough swab samples were excluded from metagenomic analysis, as research shows these do not confer correct diagnostic value for the lower airways (31). Processing and sequencing of sputum samples was performed by Integrated Microbiome Resource, Halifax, Canada following standard protocol for metagenomic sequencing (32, 33). In short, DNA was extracted from the sputum samples using the QIAGEN PowerFecal DNA Kit. The metagenomic library was prepared using the Illumina Nextera Flex (now “DNA Prep”) kit. Samples were tagmented and subsequently amplified using 12 PCR cycles. Purification was done by using beads. Samples were quantified using the Invitrogen Quant-iT dsDNA HS Assay Kit. Samples were pooled and sequenced by use of Illumina NextSeq 2000 using 150 + 150 bp paired-end chemistry.

### Phenotypic antibiotic susceptibility testing

Antibiotic susceptibility testing was done at the clinical microbiology lab, Amsterdam UMC, location AMC, Amsterdam, the Netherlands, as part of routine patient care (6) and following a standard internal operating procedure. In short, sputum samples were inoculated by following the standard clinical procedures for CF inoculation (Table S1) (34). If a bacterium was detected in these specific growth conditions, it was tested for antibiotic susceptibility. Antibiotic susceptibility was tested according to hospital policy following EUCAST criteria, by determination of the minimum inhibitory concentration and/or zone of inhibition in a disk diffusion experiment (35). Results from antibiotic susceptibility testing could be (i) susceptible, (ii) susceptible, increased exposure, or (iii) resistant. For the comparative analysis of phenotypic antibiotic susceptibility testing and the metagenomic approach, susceptible, increased exposure was grouped with resistant. It is common for diagnostic purposes to test identified bacteria for multiple specific antibiotics which belong to the same antibiotic class. If at least one specific antibiotic in an antibiotic class shows resistance, the bacterium is considered resistant. Different bacteria are tested for the same antibiotics. For the resistome analysis, if at least one bacterium showed resistance to a certain antibiotic class, all the bacteria in the sample were considered resistant. For *P. aeruginosa,* it is common practice to test multiple colonies for antibiotic resistance. If at least one of these colonies showed resistance, all colonies were considered resistant. These results are all pooled together since the antibiotic resistance genes analysis also pools the entire sample together.

### Bioinformatics processing and antibiotic-resistant genes identification

The quality of the metagenomics data was checked by FastQC version 0.11.8 (http://www.bioinformatics.babraham.ac.uk/projects/fastqc/). Next, sequencing reads were aligned to the human genome with Bowtie2 version 2.4.2, using human genome reference GRCh38 (36). This was used to remove the host-associated DNA from the metagenomic reads by KneadData v0.7.4 (https://huttenhower.sph.harvard.edu/kneaddata). Lastly, short reads (<35 base pairs) and low-quality reads (<15 Q) were removed by Trimmomatic v0.39 (37).

**Fig 1:**
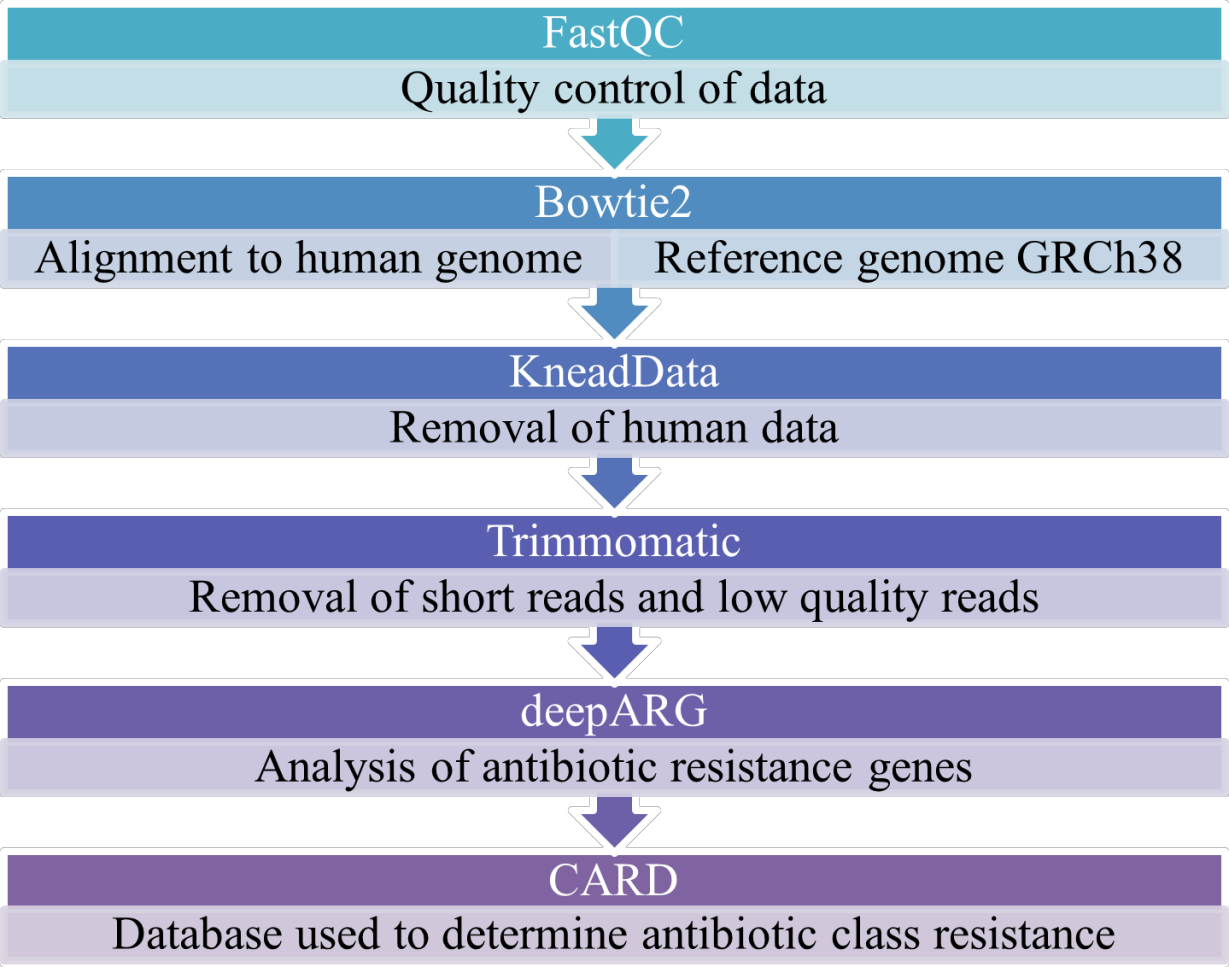
Overview of the bioinformatics processing and antibiotic resistance genes identification.

Analysis of antibiotic resistance genes was done by the use of the short reads pipeline of deepARG version 2.0 (38). DeepARG is a deep learning tool that has been developed using “dissimilarity-based classification.” In this approach, ARGs from the Comprehensive Antibiotic Resistance Database (CARD) (39) and the antibiotic resistance genes database (40) were used as reference ARGs, and ARGs from the Universal Protein Resource (UniProt) database (41) were used to train and validate deepARG. Classification is done by determining how dissimilar an ARG is from the reference ARGs. Specifically, ARGs from the UniProt database were split into short reads for the short reads pipeline. Furthermore, 70% of ARGs were used for the training data set, and 30% of ARGs were used to validate the model (38). For analysis in this work, the following parameters were used: gene_coverage 1%, deeparg_identity 80%, deeparg_probability 95%, bowtie_16 s_identity 100%. CARD was used to determine to which antibiotic class the identified ARGs confer resistance (39). An overview of the bioinformatics processing and ARGs identification can be found in Fig. 1. As described in the supplementary materials of Neerincx et al. (30), no detectable reads were retrieved from included negative control (30).

### Statistical analysis

Data were included if both the metagenomics data and the result from the antibiotic susceptibility testing were available for the sputum sample. Each visit from each patient was considered a separate, independent sample. Antibiotic classes for which the sample size was less than 3 were not analyzed due to low power. Obtained ARGs’ gene_coverage from the deepARG analysis was used as a predictor for the lab phenotypic antibiotic susceptibility testing. The gene coverage is the fraction of the sample gene (compared to the full reference gene) that needs to be present in order to consider it as a positive read of an antibiotic resistance gene. Statistical analyses of the data were performed in R 4.0.5 via the RStudio interface version 1.4.1106, using the pROC 1.18.0 package (42). The prediction performance was assessed using the area under the curve-receiver operating characteristics (AUCROCs) with reporting the 95% confidence intervals (95% CIs). In addition, the Youden index was used to detect optimum threshold gene coverage from the AUCROCs to maximize the sensitivity and specificity, as this index is most scientifically validated (43).

## RESULTS

### Sputum samples

In total, from 97 visits, 73 sputum samples were collected for routine clinical microbial detection from 18 different patients. In other cases, cough swabs were obtained. In 69 cases from the 73 sputum samples, it was possible for the patient to produce a sputum sample for metagenomic sequencing, coming from 16 different patients. One patient stopped the use of lumacaftor/ivacaftor after visit 3 and was not further included in the study (30). Another patient routinely visited the hospital only four times during the study duration. Complete details on the amount of patient visits and samples taken can be found in Table S2.

### Microbial detection

**Fig 2:**
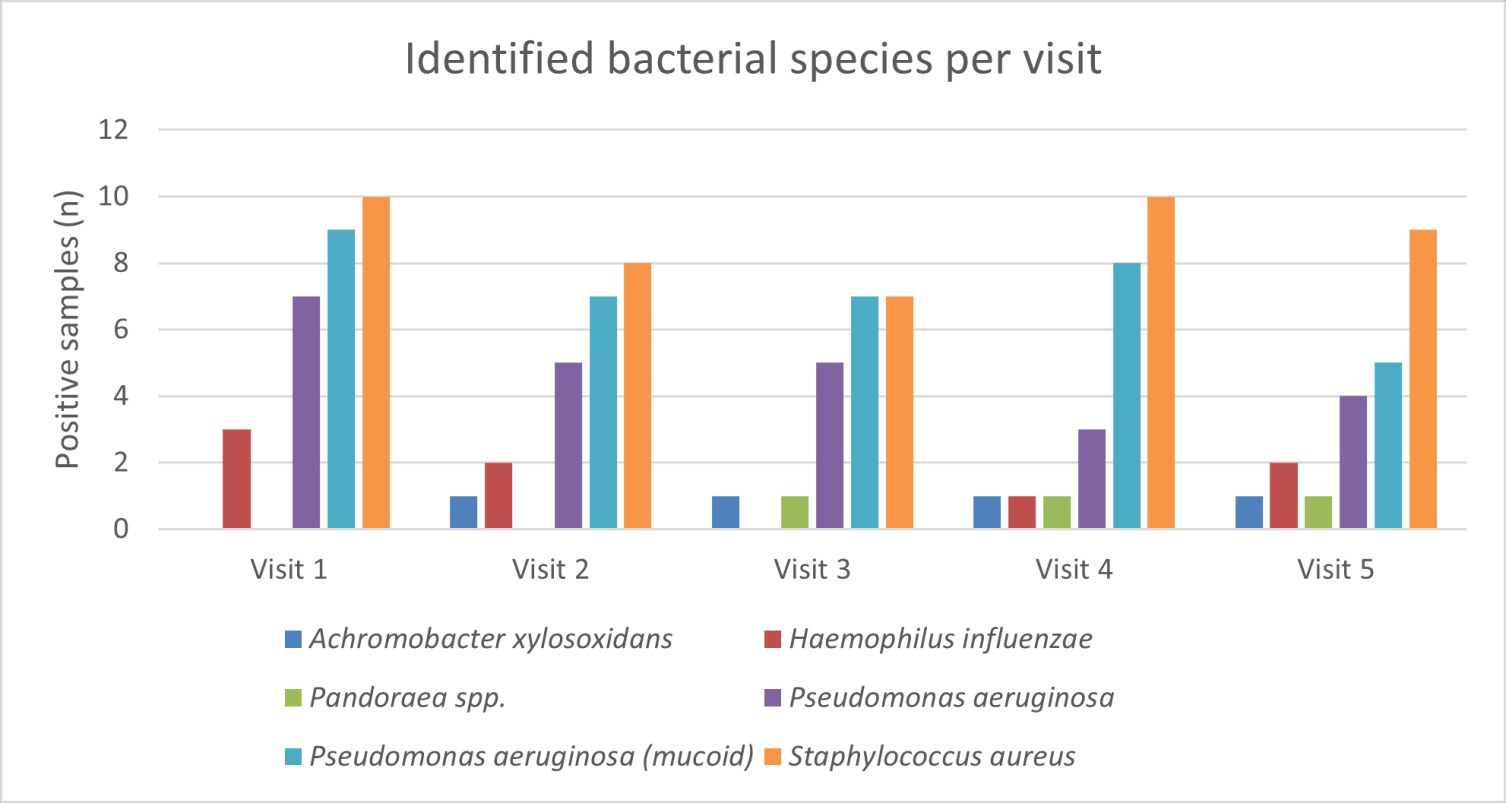
Identified bacterial species per visit in sputum samples. Each patient visited the hospital five times. For each sputum sample, bacteria were identified as part of routine clinical care. The following bacteria were detected in the sputum samples: *Achromobacter xylosoxidan*s (dark blue), *Haemophilus influenza* (red), *Pandoraea* spp. (green), *Pseudomonas aeruginosa* (non-mucoid [purple]) and mucoid type [light blue]), and *Staphylococcus aureus* (orange).

Inoculation of the sputum samples showed microbial growth in 71 of 72 samples. This includes bacterial growth (*n* = 68) as well as fungal growth (*n* = 29). The following bacteria were detected: *Achromobacter xylosoxidans* (*n* = 4), *Haemophilus influenzae* (*n* = 8), *Pandoraea* spp. (*n* = 3), *P. aeruginosa* (*n* = 42; non-mucoid [*n* = 24] and mucoid [*n* = 36]) (18 cases showed infection with both subtypes), and *S. aureus* (*n* = 44) (Fig. 2). All cases of fungal growth were *Aspergillus fumigatus* (data not shown). Details of the detected bacteria per patient are shown in Table S3.

### Antibiotic susceptibility of sputum samples

**Fig 3:**
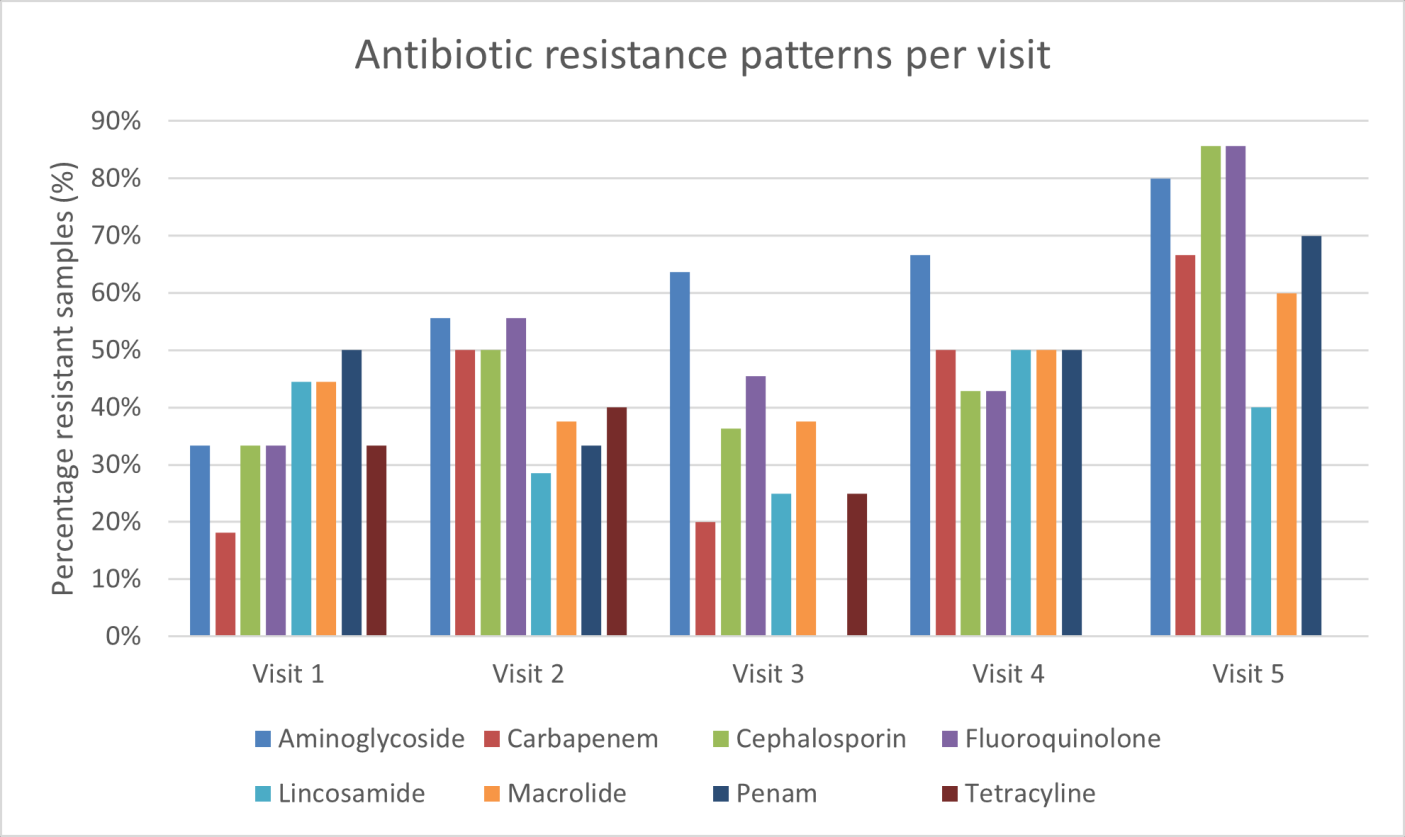
Antibiotic resistance in sputum samples per antibiotic class per visit. The detected bacteria in the sputum sample were tested for antibiotic susceptibility. For each antibiotic class, the percentage of antibiotic-resistant samples is shown. Resistance is shown for eight antibiotic classes: aminoglycoside (blue), carbapenem (red), cephalosporin (green), fluoroquinolone (purple), lincosamide (light blue), macrolide (orange), penam (dark blue), and tetracycline (brown).

From 72 sputum samples, 68 samples were suited for ABS testing in routine care. Two samples showed no bacterial growth. For one sputum sample, it was not possible to determine the antibiotic susceptibility due to overgrowth of *A. fumigatus*. This sample was suited for metagenomic sequencing and deepARG analysis but has not been included in further analysis. For one sputum sample, no antibiotic susceptibility data were available. The results are summarized in antibiotic class per patient per visit, as antibiotic resistance genes are also analyzed per antibiotic class instead of a specific antibiotic. The following antibiotic classes were tested: aminoglycosides (*n* = 43; susceptible [S] = 19, resistant [R] = 24), carbapenems (*n* = 39; S = 19, R = 15, intermediate [I] = 5), cephalosporins (*n* = 47; S = 25, R = 22), fluoroquinolones (*n* = 46; S = 21, R = 24, I = 1), lincosamides (*n* = 43; S = 27, R = 16), macrolides (*n* = 44; S = 25, R = 19), penams (*n* = 44; S = 25, R = 19), and tetracyclines (*n* = 20; S = 15, R = 5) (Fig. 3). Results of antibiotic susceptibility testing per patient can be found in Table S4.

### Sample resistome prediction

**Fig 4:**
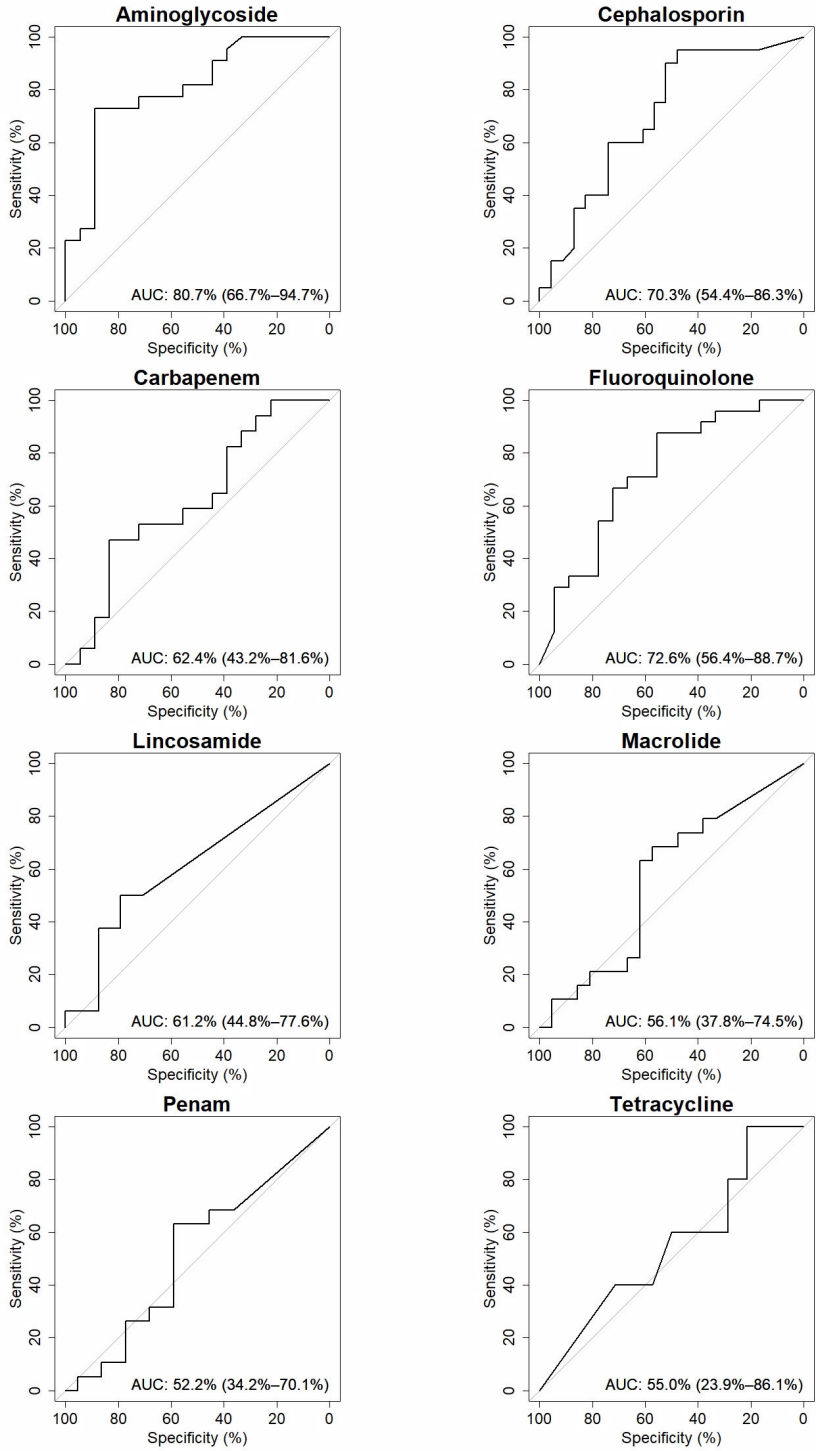
Separate AUCROC curves for each antibiotic class. For each ROC curve, the area under the curve is calculated, with 95% confidence intervals in brackets.

DeepARG was used to identify antibiotic resistance genes for 68 metagenomics sputum samples (38). The most commonly occurring ARGs are shown in Table 1. For each antibiotic class, the performance of deepARG is visualized in area under the curve-receiver operating characteristics curves (Fig. 4). Aminoglycoside had the highest AUCROC of 80.69% (95% CI 66.71–94.66) (*n* = 40). For both fluoroquinolone (72.57% [95% CI 56.4–88.74]) (*n* = 42) and cephalosporin (70.33% [95% CI 54.36–86.29]) (*n* = 43), deepARG also showed a statistically significant performance. For the other antibiotic classes, the results were not statistically significant: carbapenem 62.42% (95% CI 43.24–81.59) (*n* = 35), lincosamide 61.20% (95% CI 44.76–77.63) (*n* = 40), macrolide 56.14% (95% CI 37.76–74.52) (*n* = 40), penam 52.15% (95% CI 34.21–70.09) (*n* = 41), and tetracycline 55% (95% CI 23.86–86.14) (*n* = 19). With the use of the Youden index, the optimal gene coverage threshold for each antibiotic class was determined (Table 2). For aminoglycoside, cephalosporin, and fluoroquinolone, this value was 0.33, 0.18, and 0.31, respectively. Using the calculated threshold, the sensitivity, specificity, negative predictive value (NPV), and positive predictive value (PPV) for each antibiotic class were determined(Table 2). The sensitivity for aminoglycoside, cephalosporin, and fluoroquinolone was 72.73%, 95.00%, and 87.50%, respectively, while the corresponding specificities were 88.89%, 47.83%, and 55.56%.

**TABLE 1:** Overview of most common occurring antibiotic resistance genes in this study^*a*^

Gene	Resistance	Organism	Samples (*n*)
rpoB2	Rifamycin	*Nocardia farcinica*	38
MexI	Multidrug	*P. aeruginosa*	34
MuxB	Multidrug	*P. aeruginosa*, *P. fluorescens*	34
MexF	Multidrug	*P. aeruginosa*	33
arnA	Peptide	*P. aeruginosa*	32
mexN	Phenicol	*P. aeruginosa*, *P. fluorescens*	32
MexD	Multidrug	*P. aeruginosa*, *P. fluorescens*	31
MuxC	Multidrug	*P. aeruginosa*	31
TriC	Disinfecting agents and antiseptics	*P. aeruginosa*	31
MexB	Multidrug	*P. aeruginosa*	30
MexW	Multidrug	*P. aeruginosa*	30

^*a*^ Shown are the ARGs, the resistance it causes, from which organisms they originate and in how many samples these genes were found. This information was retrieved from the CARD (39).

**TABLE 2:** Values determined using the AUCROC curves for each antibiotic class^*a*^

Antibiotic class	Number of samples	Percentage resistance	Threshold gene coverage	Sensitivity	Specificity	NPV	PPV
Aminoglycoside	41	56.10	0.33	72.73	88.89	72.73	88.89
Carbapenem	36	52.78	0.54	47.06	83.33	62.50	72.73
Cephalosporin	44	47.73	0.18	95.00	47.83	91.67	61.29
Fluoroquinolone	43	55.81	0.31	87.50	55.56	76.92	72.41
Lincosamide	40	40.00	0.11	50.00	79.17	70.37	61.54
Macrolide	40	47.50	0.18	68.42	57.14	66.67	59.09
Penam	41	46.34	0.12	63.16	59.09	65.00	57.14
Tetracycline	19	26.32	0.44	100.0	21.43	100.0	31.25

^*a*^ For each antibiotic class, shown are the number of samples, the percentage of resistant samples, threshold gene coverage, and the corresponding sensitivity, specificity, negative predictive value, and positive predictive value in percentage. The threshold gene coverage is calculated based on the Youden index. Note that tetracycline had a considerably lower sample size than other classes, as well a low number of cases of resistance according to the antibiotic susceptibility testing obtained during routine clinical care.

### Species resistome prediction

**Fig 5:**
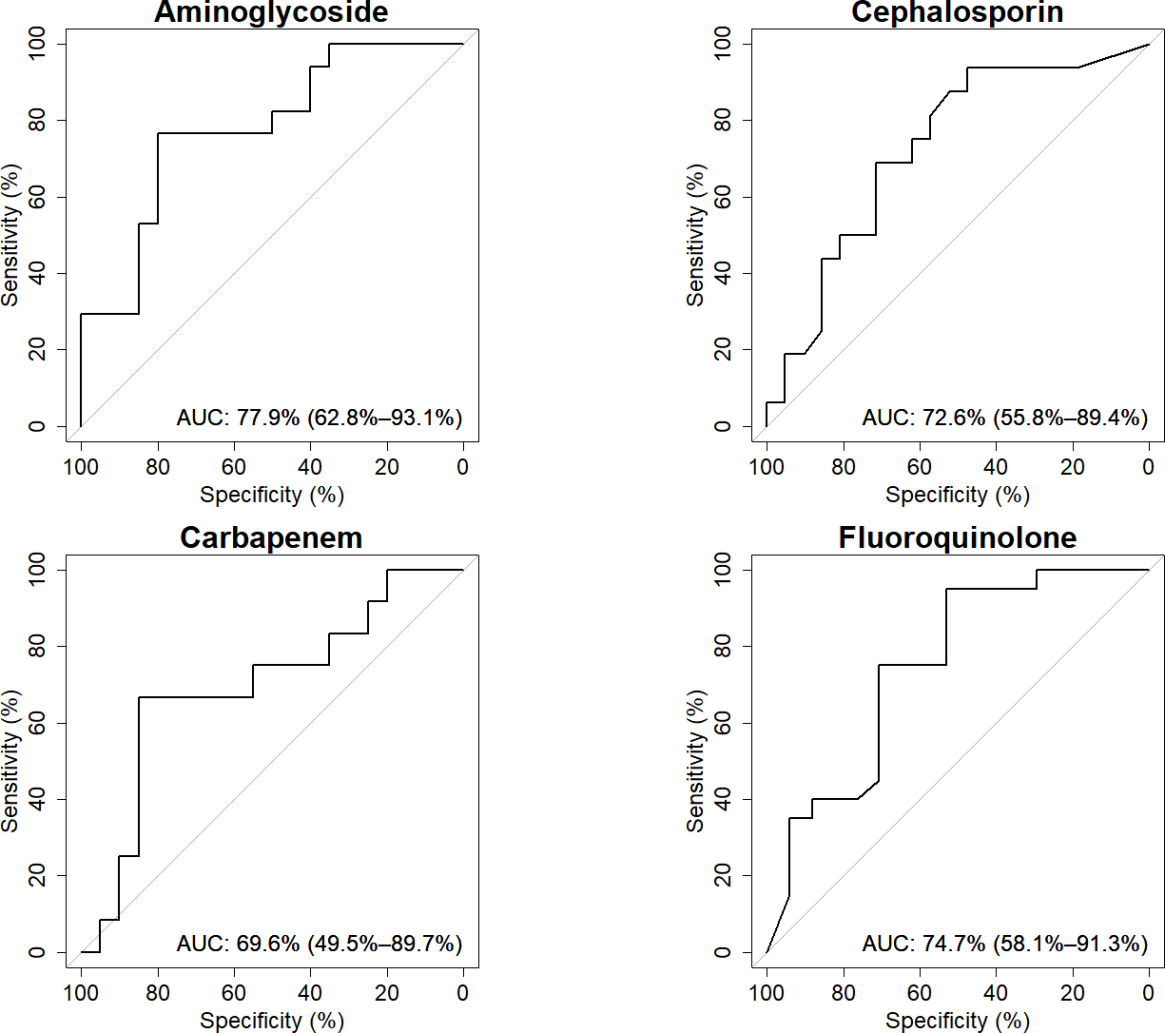
Separate AUCROC curves for each antibiotic class in *P. aeruginosa*. AUC values are calculated for each ROC curve, with 95% confidence intervals indicated in brackets.

DeepARG was applied to determine the species-specific resistome per antibiotic class. For *P. aeruginosa*, data were available for the antibiotic classes aminoglycosides, fluoroquinolones, cephalosporins, and carbapenems. For each class, the performance of deepARG was evaluated by AUCROC curves (Fig. 5). Similar to the sample resistome prediction, significant performance was obtained for the classes aminoglycosides (77.94% [95% CI 62.76–93.12]) (*n* = 37), fluoroquinolones (74.71% [95% CI 58.08–91.33]) (*n* = 37), and cephalosporin (72.62% [95% CI 55.79–89.45]) (*n* = 37). Carbapenem did not reach statistical significance (69.58% [95% CI 49.51–89.65]) (*n* = 32). Optimal gene coverage thresholds for each antibiotic class were identified using the Youden index (Table 3). For aminoglycoside, cephalosporin, and fluoroquinolone, the thresholds were 0.33, 0.17, and 0.31, respectively. Based on these thresholds, the sensitivity, specificity, NPV, and PPV were calculated (Table 3). Sensitivities for aminoglycoside, cephalosporin, and fluoroquinolone were 76.47%, 93.75%, and 95.00%, respectively, with corresponding specificities of 80.00%, 47.62%, and 52.94%.

**TABLE 3:** Values determined using the AUCROC curves for each antibiotic class in *P. aeruginosa^a^*

Antibiotic class	Number of samples	Percentage resistance	Threshold gene coverage	Sensitivity	Specificity	NPV	PPV
Aminoglycoside	37	45.95	0.33	76.47	80.00	80.00	76.47
Carbapenem	32	37.50	0.54	66.67	85.00	80.95	72.73
Cephalosporin	37	43.24	0.17	93.75	47.62	90.91	57.69
Fluoroquinolone	37	54.05	0.31	95.00	52.94	90.00	70.37

^*a*^ Detected ARGs were cross-referenced with the CARD (39). Only ARGs derived from *P. aeruginosa* were used to calculate AUCROC curves. For each antibiotic class, shown are the number of samples, the percentage of resistant samples, threshold gene coverage, and the corresponding sensitivity, specificity, NPV, and PPV in percentage. The threshold gene coverage is calculated based on the Youden index.

For *S. aureus*, per antibiotic class, in only three to five samples, relevant ARGs were detected, and performance could not be determined. As for *A. xylosoxidans*, *H. influenzae,* and *Pandoraea* spp., the sample size was 8 or less; these have been excluded from analysis due to low statistical power.

## DISCUSSION

This study aimed to explore whether the detection of antibiotic resistance genes in sputum samples of adult people with CF, using a metagenomics approach, could be used to determine the antibiotic susceptibility phenotype. Our findings suggest that the detection of ARGs can be used, at least, for the antibiotic classes aminoglycoside, cephalosporin, and fluoroquinolone. For the antibiotic classes carbapenem, lincosamide, macrolide, penam, and tetracycline, the results were not statistically significant. Furthermore, similar results were obtained when focusing on the antibiotic susceptibility phenotype of *P. aeruginosa*-positive infections alone, including a slight improvement for the antibiotic class carbapenem, thereby almost reaching statistical significance. Using antibiotic class-specific cut-offs for positive reads of ARGs, a metagenomics approach offers a culture-independent and more time-efficient manner to determine ABS for certain antibiotic classes for sputum samples of adult people with CF.

To the best of our knowledge, the current study is one of the first to explore the potential of artificial intelligence tools to detect ARGs in a clinical diagnostic setting (27). With growing interest in the human microbiome, the amount of bioinformatics tools to analyze microbiome data are increasing (44, 45). Several tools to detect ABS have been developed (46). Although some of these tools have been developed with the clinic in mind, the use of them as a diagnostic instrument is currently underdeveloped (28, 47). Positive results achieved in research settings do not always translate into similarly high-quality results in clinical settings. For instance, the performance of these tools is partly dependent on the chosen training data set. This data set can, for instance, be influenced by the time of sample collection or the geographical location where the sample was taken. This can lead to incorrect representation of clinical data. It can result in sampling bias, such as over-representation of certain bacterial species or under-representation of antibiotic class resistance (48, 49). The further optimization of such data sets can improve the performance of tools that detect ABS. There can also be little data available to train a new tool, for instance, on ARGs for novel antibiotics (50), although new bioinformatics tools are being developed to handle this (51). Another challenge is antibiotic resistance driven by unknown genes, which currently cannot be identified by ABS detection tools (27), although advances in this field are ongoing (52). Currently, classical culture-dependent ABS testing remains the gold standard in clinic (14).

A strong point of this study is the utilization of an artificial intelligence tool, more specifically deep learning, to detect antibiotic-resistant genes. Most tools that determine ARGs use a best-hit approach, using strict cut-offs to determine whether a sequence read is an ARG. DeepARG is not dependent on strict cut-off points, thereby reducing the likelihood of false negatives and thus offering a more inclusive analysis of ARGs (38). DeepARG uses three big databases to determine antibiotic-resistant genes, two of which are continuously updated, namely the Comprehensive Antibiotic Resistance Database (39) and Universal Protein Resource database (41). The use of a bioinformatics tool, in general, offers the possibility to determine antibiotic-resistant genes culture independently, thereby offering the possibility to determine ABS for less well-known bacteria. This study used real-life clinical data obtained from adult people with CF across a time period of 1 year. Apart from a similar initiation of the use of lumacaftor/ivacaftor (30), patients were not selected on the basis of disease state or other medications. This strengthens our data set as a representation of the CF population with a homozygous Phe508del mutation, the most common CF-causing mutation (53). Furthermore, these results might be extendable to other pulmonary infection cases, such as pneumonia, or other diseases, such as bronchiectasis and primary ciliary dyskinesia.

This study also has some limitations. First, deepARG is not able to detect ARGs resulting from single-nucleotide polymorphisms (SNPs) (38). SNPs are very common genetic mutations that also confer antibiotic resistance. For instance, several studies reported numerous SNPs to cause antibiotic resistance in *P. aeruginosa* (54–56). Second, no ARGs were detected for *S. aureus*. As *S. aureus* is an important source of lung infection in people with CF (4), it is important to be able to predict the antibiotic susceptibility of *S. aureus*-positive samples as well. The lack of ARGs detection for *S. aureus* is likely due to the sequencing quality, resulting in issues with isolating DNA from Gram-positive bacteria due to their rigid cell wall (57). Third, due to the low sample size, internal validation of findings was not possible. The sequencing depth and sample size also did not allow for performing analysis on drug level rather than class level. Fourth, the patients in this study all received the same medication (lumacaftor/ivacaftor), which ameliorated their symptoms (30, 58). This made it difficult for some of the patients to produce sputum, and subsequently, cough swab samples were taken, which resulted in a loss of follow-up sputum samples. Research has shown that cough swab samples do not accurately represent pathogens from the lower airways compared to a sputum sample (31). Due to new medications such as ivacaftor/tezacaftor/elexacaftor, patients show strongly improved symptoms (59). The amount of infections lowers drastically, and the production of sputum is diminished (60), although research has shown that even with the use of new medications, chronic *P. aeruginosa* infections persist (61). It should be investigated whether the current approach can also be effective with use of, e.g., cough swabs or nasal washes. It is important to note that the lack of sputum samples also is challenging for phenotypic ABS testing. Fifth, it is also important to note that due to the nature of the study (30), the most severely ill people with CF have not been included. Lastly, as with the current data set no internal validation is possible; it is essential to use an external data set of people with CF to validate the results.

Although metagenomics offers the possibility to develop relatively fast protocols compared to traditional cultures (25), it requires more hands-on time and resources, and therefore, this could add to the costs and facilities required to apply this technology in clinical practice. Also, as with any bioinformatics tool, the performance of deepARG is also dependent on the training data sets, which can be biased due to a number of factors as introduced before. These biases can influence the accuracy of results obtained when not relying on strict cut-offs. Another important note is that the *in vitro* findings of ABS phenotypes do not necessarily correspond with the *in vivo* reaction of the patient (21). The lungs comprise a community of bacteria and other microorganisms. These different microorganisms influence each other and are in an equilibrium with each other (62). For instance, one species can acquire resistance to beta-lactam antibiotics through the production of beta-lactamases by another species (63). These environmental conditions cannot be perfectly mimicked in *in vitro* conditions. A general challenge in the use of metagenomics is the distinction between a bacterium as the cause of infection and as commonly present in the lung microbiota (64, 65). This limitation is also applicable when predicting the presence of ARGs, as ARGs can also be present in the background without involvement in the current infection (66), although it is not expected that the multidrug ARGs found in this study are present in non-infected individuals (67). Important to note is that bacteria can also be intrinsically resistant to certain antibiotics, which will not be detected when analyzing for ARGs, using tools such as DeepARG (68).

Antibiotic resistance is a growing problem worldwide, especially so for patients strongly dependent on antibiotics, such as people with CF (7). For instance, a core macrolide resistance in airways of patients with chronic lung infections has been reported (67). Macrolide antibiotics have been proven to ameliorate symptoms for people with CF, including less pulmonary exacerbations (69). However, Southern et al. (69) already reported concerns about growing resistance to macrolide antibiotics (69). Moreover, genes conferring aminoglycoside resistance have been found across continents, suggesting a worldwide spread of the same genes (70). Aminoglycosides are often prescribed to people with CF with chronic *P. aeruginosa* infections (71, 72), and resistance of *P. aeruginosa* to aminoglycosides has been detected in people with CF (73). The existing problem of aminoglycoside resistance shows the valuable use of our strong prediction results for aminoglycosides. In general, hypermutator strains of *P. aeruginosa*, which quickly develop multidrug resistance, have been observed in people with CF (74). Another bacterium observed in chronic lung infections of people with CF with increasing antibiotic resistance is *Achromobacter* species (75). Multidrug-resistant bacterial lung infections in people with CF are an increasing problem.

Timely determination of ABS is crucial, as lung infections are the leading cause for the mortality and morbidity of CF (1–3). Current methods to determine ABS are time consuming and culture dependent. Some bacteria take days to weeks to grow in *in vitro* circumstances, and different bacteria need different growth conditions (13). As a consequence, patients are often prescribed broad-spectrum antibiotics while awaiting the results of ABS testing (11). Our data also showed that for one sample, it was not possible to determine ABS due to overgrowth of fungi (*A. fumigatus*), while it was possible to predict ARGs in this sample using the metagenomics approach, suggesting the potential usefulness of this technique to screen for antibiotic susceptibility in cases where bacteria are difficult to culture. A specific subset of bacterial cells, the so-called persister cells, will likely not even be detected by general ABS testing. These dormant, drug-tolerant microbial cells are suggested to be important contributors to chronic *P. aeruginosa* lung infections in people with CF (76). Previous research has shown that a highly optimized procedure for the detection of ARGs can be executed in just 6 hours (25). The use of metagenomics sequencing in combination with artificial intelligence tools for predicting antibiotic resistance might save crucial time in clinical care to determine the appropriate antibiotic to prescribe to the CF patient, as well as in other serious pulmonary infections where timely interventions with antibiotics are crucial, e.g., in bronchiectasis (77, 78), primary ciliary dyskinesia (79), and pneumonia in immunocompromised individuals (80). This will result in better clinical care, as well as the prevention of prescribing less effective antibiotics, which also results in less contribution to the ongoing problem of antibiotic resistance in general.

In conclusion, our results suggest that the combination of metagenomics and artificial intelligence tools, such as deepARG, can be useful in the clinical detection of ABS, at least for certain antibiotic classes. It is important to prescribe antibiotics to people with CF in an effective manner, both considering time and resistance, to prevent chronic infections, offer adequate treatment for the existing infection, and prevent the spread of antibiotic resistance. Bioinformatics tools can aid in this process by complementing the findings of general ABS testing to prevent prescribing antibiotics to which the patient is already resistant, as well as offering a time-efficient and culture-independent way of determining ABS.

## Data Availability

The metagenomic sequencing data have been deposited in the European Nucleotide Archive (ENA) at EMBL-EBI under accession number PRJEB96952.

## SUPPLEMENTAL MATERIAL

Supplemental material spectrum.02299-24-s0001.docx Tables S1 to S4.

ASM does not own the copyrights to Supplemental Material that may be linked to, or accessed through, an article. The authors have granted ASM a non-exclusive, world-wide license to publish the Supplemental Material files. Please contact the corresponding author directly for reuse.
