# Supplementary material for: Metagenomics approach to predict antibiotic resistance genes in sputum samples of adult people with cystic fibrosis: a pilot study
Source: Microbiol Spectr. 2025 Nov 5;13(12):e02299-24. doi: 10.1128/spectrum.02299-24 (PMC12671179; doi:10.1128/spectrum.02299-24)
Supplement: Supplemental material — Tables S1 to S4. [file spectrum.02299-24-s0001.docx]

**Table S1 Culture medias for sputum samples obtained from people with CF following the standard clinical procedure.** Procedure based on Laboratory Standards for Processing Microbiological Samples from People with Cystic Fibrosis (1).

| Media | Temperature | Growth condition | Duration |
| --- | --- | --- | --- |
| Chocolat agar polyvitex (biomérieux) | 35-37 °C | 5% CO_2_ | 3 days |
| Columbia agar with 5% sheep blood (biomérieux) | 35-37 °C | 5% CO_2_ | 3 days |
| Mac conkey agar (biomérieux) | 35-37 °C | O_2_ (air) | 3 days |
| Chromid® s. Aureus elite agar (saide) (biomérieux) | 35-37 °C | O_2_ (air) | 2 days |
| Burkholderia cepacia selective agar (biomérieux) | 35-37 °C | O_2_ (air) | 3 days |
| Mueller hinton e agar (biomérieux) | 35-37 °C | O_2_ (air) | 2 days |

**Table S2** **Type of microbial samples collected from patients during five routine visits**. Patients were asked to cough deeply to produce sputum. If this was not possible, a cough swab was taken. These results were obtained during routine clinical microbial detection. ♦ indicates that the sample showed no microbial growth. ◦ indicates that the sample only showed fungal growth. ◊ indicates that no sample was taken for metagenomics sequencing. An x indicates that the patient stopped the use of lumacaftor/ivacaftor and was not further included in the study. An o indicates that this visit did not occur.

|  | Visit 1 | Visit 2 | Visit 3 | Visit 4 | Visit 5 |
| --- | --- | --- | --- | --- | --- |
| Patient 1 | Sputum | Sputum ◊ | Sputum | Sputum | Sputum |
| Patient 2 | Sputum | Sputum | Sputum | Sputum | Sputum |
| Patient 3 | Cough swab ♦ | Sputum ◊ | Cough swab ♦ | Cough swab ♦ | Cough swab ♦ |
| Patient 4 | Cough swab | Sputum | Sputum | Sputum | Sputum |
| Patient 5 | Sputum | Sputum | Sputum | Sputum | Sputum |
| Patient 6 | Cough swab ♦ | Cough swab | Cough swab ♦ | Cough swab ♦ | Cough swab ♦ |
| Patient 7 | Sputum | Sputum | Sputum ♦ | Sputum | Sputum |
| Patient 8 | Sputum | Sputum | Sputum ◊ | x | x |
| Patient 9 | Sputum | Sputum | Sputum | Sputum | Sputum |
| Patient 10 | Sputum | Sputum | Sputum ◊ | Sputum | Sputum |
| Patient 11 | Sputum | Sputum | Sputum | Sputum | Sputum |
| Patient 12 | Sputum | Sputum | Sputum | Sputum | Sputum |
| Patient 13 | Sputum | Cough swab ♦ | Cough swab | Sputum ◊ | o |
| Patient 14 | Sputum | Cough swab ♦ | Cough swab | Cough swab ♦ | Cough swab ♦ |
| Patient 15 | Sputum | Sputum | Sputum | Sputum | Sputum ◦ |
| Patient 16 | Sputum | Sputum | Sputum | Sputum | Sputum |
| Patient 17 | Sputum | Sputum | Sputum | Sputum | Sputum |
| Patient 18 | Sputum | Sputum | Sputum | Sputum | Sputum |
| Patient 19 | Cough swab ♦ | Cough swab | Cough swab ♦ | Cough swab | Cough swab ♦ |
| Patient 20 | Cough swab ♦ | Cough swab | Sputum ◊ | Cough swab | Cough swab ♦ |

**Table S3** **Bacteria detected in sputum samples during routine clinical care per patient**. Green indicates that the bacterium was detected, blue indicates that the sample showed no bacterial/fungal growth at all, white indicates that the sample showed no growth of that specific bacterium, black indicates that there was no sputum sample available for that visit.


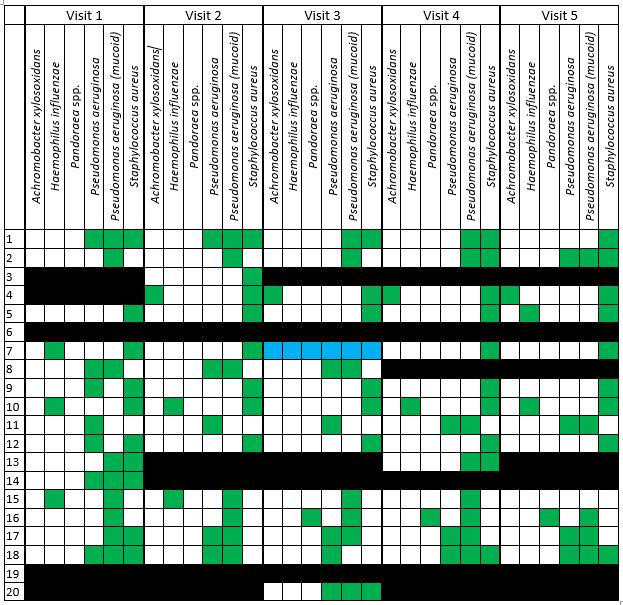


**
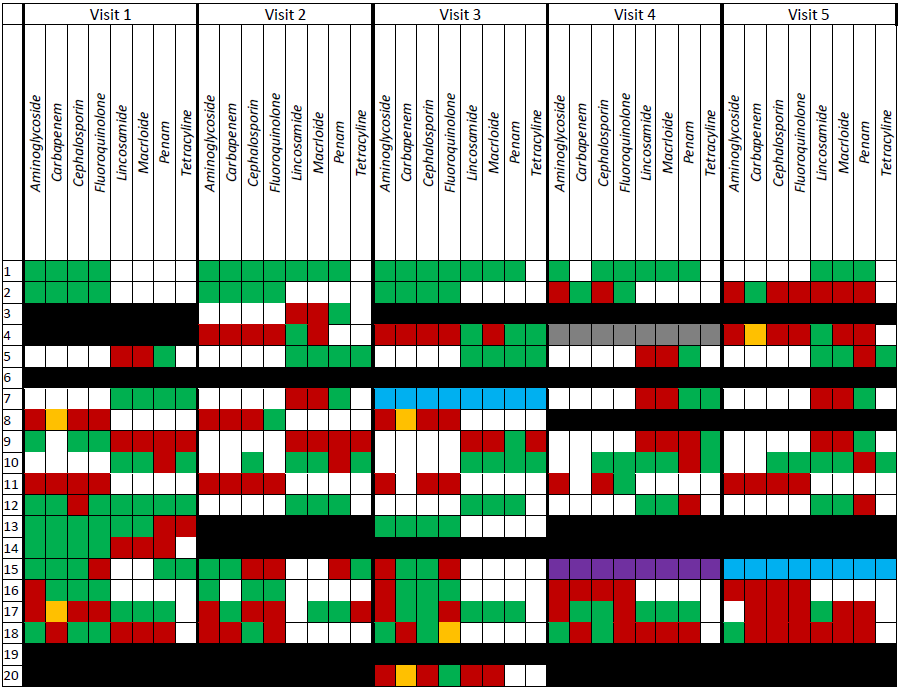
Table S4** **Results from antibiotic susceptibility testing of sputum for twenty patients over five visits.** If at least one of the tested antibiotics in an antibiotic class showed resistance, the sample was considered resistant. If at least one of the tested bacteria showed resistance to an antibiotic class, the sample was considered resistant. If at least one colony of *P. aeruginosa* showed resistance, the sample was considered resistant. Red indicates Resistance, green indicates Susceptibility, orange indicates Intermediate susceptibility. Blue indicates no bacterial growth. Purple indicates that antibiotic susceptibility testing was not possible due to overgrowth of A. fumigatus. Grey indicates that the antibiotic susceptibility result was not available. Black indicates that there was no sputum sample available. White indicates that this antibiotic class was not tested for this sputum sample.

**References:**

1. The UK Cystic Fibrosis Trust Microbiology Laboratory Standards Working Group. 2022. Laboratory Standards for Processing Microbiological Samples from People with Cystic Fibrosis. Cystic Fibrosis Trust,
